# A Case of Successful Neurological Critical Care for the Very-High-Risk Refeeding Syndrome With Central Nervous System Disorders

**DOI:** 10.7759/cureus.94574

**Published:** 2025-10-14

**Authors:** Yojiro Kashimura, Masaki Mochida, Tomoyoshi Tamura, Koichi Ueno

**Affiliations:** 1 Department of Neuroscience, Kawasaki Municipal Hospital, Kawasaki, JPN; 2 Department of Emergency and Critical Care Medicine, Kawasaki Municipal Hospital, Kawasaki, JPN; 3 Department of Emergency and Critical Care Medicine, Keio University School of Medicine, Tokyo, JPN

**Keywords:** central nervous system, neurocritical care, neurology, nutrition, refeeding syndrome

## Abstract

Refeeding syndrome potentially causes death from refeeding-induced central nervous system disorders, such as hypoglycemic encephalopathy, Wernicke encephalopathy, and hypophosphatemic encephalopathy. However, there is no established management for malnourished patients with this refeeding-induced central nervous system syndrome. We report a case of successful neurological critical care for refeeding-induced central nervous system syndrome. A 15-year-old female with anorexia nervosa presented to our hospital after fainting. Her body mass index (BMI) was 8.8 kg/m². Upon arrival, her Glasgow Coma Scale was E4V4M6, plasma glucose was 25 mg/dL, aspartate aminotransferase was 1393 U/L, and alanine transaminase was 1368 U/L. We started a continuous 5% dextrose. However, her Glasgow Coma Scale dropped to E4V1M1 1 hour later, and her plasma glucose was 28 mg/dL. We delivered her to the neurological critical care unit and started critical nutritional management. Her laboratory data had gradually improved. On day five from admission, her Glasgow Coma Scale had improved to G4V4M6. On day 15. She was transferred to the psychiatric department. In conclusion, clinicians should recognize that refeeding-induced central nervous system syndrome is neurologically critical and life-threatening. However, our management successfully improved this patient's condition. Additionally, she gained enough weight on day 15 after admission. Our moderate-calorie nutritional management might be adequate for refeeding-induced central nervous system syndrome. However, it is a little too exaggerated to conclude that our management is safe and the best nutritional management, based only on this case. We recommend a future observational study as the next step.

## Introduction

Refeeding syndrome is a severe disease that potentially puts malnourished patients at risk of refeeding-induced central nervous system syndrome (RICNSS). Past reports demonstrated the RICNSS, such as hypoglycemic encephalopathy, Wernicke encephalopathy, seizures, hypophosphatemic encephalopathy, osmotic demyelination syndrome, and hyperammonemic encephalopathy, sometimes inflict patients with permanent neurological damage [1-6]. However, there is no definitive consensus about nutritional management for this RICNSS because there have been no robust evidence or methods to diagnose the definitive patient's condition. That is why we propose the RICNSS concept for future studies.

In addition, refeeding syndrome also causes life-threatening diseases, such as refeeding-induced hepatitis or cardiac dysfunction, which is not so rare [7]. The adequate nutritional management for such patients remains an unsolved issue. It would be an urgent problem to establish a management method for very-high-risk refeeding syndrome with RICNSS.

## Case presentation

**Figure 1:**
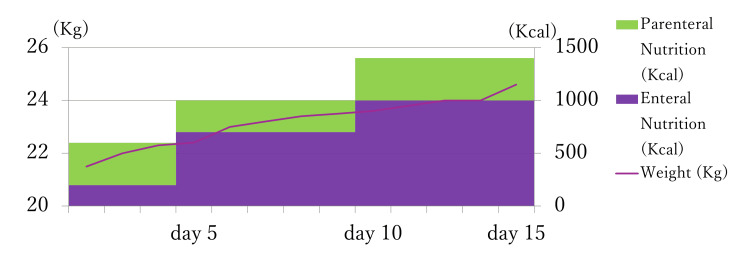
Timeline of the calorie intake and body weight The image is created by the author. Kg: Kilogram; Kcal: Kilocalorie

A 15-year-old female with anorexia nervosa presented to our hospital after fainting. Her body weight is 21.9 kg (Figure 1), and she is 5 feet 2 inches tall (BMI: 8.8 kg/m²). Upon arrival, her vital signs are as follows: Glasgow Coma Scale (GCS) is E4V4M6; temperature, 36.0℃; blood pressure (BP), 84/69 mmHg; pulse, regular 80 beats/min. She stated, “I have always been a little too heavy.” Her skin and mucous membranes are dry. The neurological findings are normal, including deep tendon reflexes and eye movements. 

**Figure 2:**
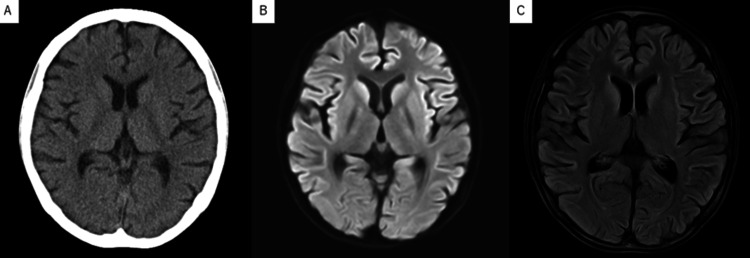
Radiologic findings of the brain (A) The computed tomography showing the generalized brain atrophy. (B) The magnetic resonance imaging (diffusion-weighted imaging) showed no findings, such as Wernicke encephalopathy. (C) The magnetic resonance imaging (fluid attenuated inversion recovery) showing the generalized brain atrophy.

Laboratory tests (Table 1) revealed plasma glucose 25 mg/dL, phosphate 3.5 mg/dL, magnesium 1.8 mg/dL, potassium 3.5 mEq/L, AST 1393 U/L, and ALT 1368 U/L. Her computed tomography showed generalized brain atrophy. We started vitamin B1 10 mg, acetated Ringer solution with 5% dextrose, and potassium supplementation 10 mEq at the emergency room. However, her GCS dropped to E4V1M1 1 hour later, and her plasma glucose was 28 mg/dL; the electroencephalography was unremarkable. We delivered her to the neurological critical care unit and inserted a central venous catheter and nasogastric tube. Initial nutrition management was intravenous 400 Kcal glucose/day, enteral 200 Kcal amino acid/day, vitamin B1 1.5 g/day, phosphate 0.6 mmol/kg/day, and magnesium 0.2 mmol/kg/day. We monitored her plasma glucose every 1 hour, blood phosphate and electrolytes every three hours, and blood ammonia, vitamin B1, vitamin B12, and magnesium every day. The phosphate dropped to 2.2 mg/dL on the next day but was corrected immediately. The other laboratory values, including plasma glucose, were managed within normal range. On day three after admission, her GCS was still impaired to E2V1M4. We took a magnetic resonance imaging scan to rule out irreversible hypoglycemic encephalopathy, Wernicke encephalopathy, and non-convulsive systemic epilepticus, but it revealed no findings other than brain atrophy (Figure 2). On the other hand, her laboratory data, such as liver enzyme levels, had gradually improved. Other laboratory data, including electrolytes and trace elements, have also improved. We increased her enteral intake by monitoring her plasma glucose level, GCS, vital signs, and oxygen saturation. On day five, her GCS scale had improved to G4V4M6. On day seven, she developed a fever, and a urinary analysis showed a bladder infection. We started levofloxacin 100 mg/day, and the infection resolved in three days. On day 11, her liver enzyme levels dropped to aspartate aminotransferase (AST) 388 U/L and alanine transaminase (ALT) 188 U/L, and her weight increased to 24.6 kg. On day 15, she took an enteral intake of 1400 kcal/day and was transferred to the psychiatric department.

**Table 1:** Initial laboratory data WBC: white blood cells; RBC: red blood cells; Hb: hemoglobin; APTT: activated partial thromboplastin time

Test	Result	Reference Range
WBC (/μL×10^3^)	9.15*	3.5-9.0
RBC (/μL×10^3^)	348*	350-555
Hb (g/dL)	11.4*	13-16
Platelets (/μL×10^4^)	21.5	15-40
APTT (sec)	24.6	24.0-39.0
Fibrinogen (mg/dL)	139.9*	200-400
Total protein (g/dL)	6.9	6.2-7.5
Albumin (g/dL)	4.7	3.0-3.6
Total bilirubin (mg/dL)	1.4	0.4-1.5
Aspartate aminotransferase (U/L)	1393*	13-30
Alanine transaminase (U/L)	1368*	13-23
Blood urea nitrogen (U/L)	31*	7.5-17.5
Creatinine (mg/dL)	0.41*	0.5-1.8
Ammonium (μg/dL)	70	30-86
Sodium (mmol/L)	139	136-147
Chlorine (mmol/L)	103	102-108
Potassium (mmol/L)	3.1*	3.4-4.7
Calcium (mg/dL)	8.1*	8.2-10.0
Phosphate (mg/dL)	3.5	2.5-4.5
Magnesium (mg/dL)	1.8	1.5-2.2
Glucose (mg/dL)	25*	73-109
C-reactive protein (mg/dL)	0.01	＜0.3

## Discussion

Refeeding syndrome is a life-threatening situation, and initial nutritional management has been recommended to be low-calorie for very-high-risk patients (5-10 kcal if BMI is under 15 kg/m², weight loss＞20% in the preceding 3-6 months, starvation for＞15 days) [7]. However, critical encephalopathy caused by refeeding syndrome is sometimes inevitable due to this low-calorie nutritional management [1-6]. The prolonged encephalopathy, such as hypophosphatemia or hypoglycemia, potentially causes an eternal neurological sequela or even death [1-6]. In addition, the mechanism of RICNSS has not been revealed at all.

Recently, studies about high-calorie nutritional management have been published. Their outcomes suggest effectiveness from the viewpoints of long-term overall survival rates, cost-effectiveness, and weight improvements [8,9]. However, the challenging point of high-calorie management is whether it would be applicable for patients with RICNSS, as in this patient. There have been no confirmatory methods that could predict or prevent RICNSS, such as hypophosphatemic encephalopathy, and therefore, it theoretically has a high risk of death due to brain phosphate consumption from high-calorie nutritional management. On the other hand, classical low-calorie nutritional management commonly causes hypoglycemic encephalopathy [2-6].

We thoroughly reviewed past studies to improve this patient's encephalopathy. Several articles have reported similar cases with this patient [1-6]. From these articles, we created an initial nutritional management suitable for RICNSS. Past reports of successful treatment for RICNSS described that refractory hypoglycemia from refeeding syndrome had resolved with 3.8-6.9 g glucose/kg/day. Furthermore, the necessary nutritional management for RICNSS is as follows: vitamin B1 1.5 g/day; phosphate 0.3-0.6 mmol/kg/day; and magnesium 0.2 mmol/kg/day, monitoring plasma glucose every one hour, blood phosphate and electrolytes every three hours, and blood ammonia, vitamin B1, vitamin B12, and magnesium every day. After reviewing these articles, we started her on 4.5 g glucose/kg/day to prevent refractory hypoglycemia and monitored her vitamin B1, phosphate, and magnesium. As a result, this patient did not experience any neurological sequelae, and her starvation-induced hepatitis did not exacerbate. We gradually increased her nutrition after four days of monitoring.

Our management successfully improved her neurological and nutritional condition. Both classical low-calorie management and recent high-calorie management might be inadequate for RICNSS. However, further evaluation is necessary in more cases to conclude that our moderate-calorie management is acceptable for RICNSS.

## Conclusions

In conclusion, clinicians should recognize that RICNSS is neurologically critical and life-threatening. However, our management successfully improved her RICNSS. Additionally, she gained enough weight on day 15 after admission. Our moderate-calorie nutritional management might be adequate for refeeding syndrome with RICNSS. However, it is a little too exaggerated to conclude that our management is safe and the best nutritional management for patients with RICNSS, based only on this case. We recommend further evaluation in more cases or a future observational study.
